# Zoledronic acid treatment impairs protein geranyl-geranylation for biological effects in prostatic cells

**DOI:** 10.1186/1471-2407-6-60

**Published:** 2006-03-15

**Authors:** M Goffinet, M Thoulouzan, A Pradines, I Lajoie-Mazenc, Carolyn Weinbaum, JC Faye, S Séronie-Vivien

**Affiliations:** 1 Inserm U563, Centre de Physiopathologie de Toulouse Purpan, Département "Innovation Thérapeutique et Oncologie Moléculaire", Institut Claudius Regaud, Toulouse, France; 2 Duke University, Medical Center Department of Pharmacology & Cancer Biology, Durham, NC, USA

## Abstract

**Background:**

Nitrogen-containing bisphosphonates (N-BPs) have been designed to inhibit osteoclast-mediated bone resorption. However, it is now accepted that part of their anti-tumor activities is related to interference with the mevalonate pathway.

**Methods:**

We investigated the effects of zoledronic acid (ZOL), on cell proliferation and protein isoprenylation in two tumoral (LnCAP, PC-3,), and one normal established (PNT1-A) prostatic cell line. To assess if inhibition of geranyl-geranylation by ZOL impairs the biological activity of RhoA GTPase, we studied the LPA-induced formation of stress fibers. The inhibitory effect of ZOL on geranyl geranyl transferase I was checked biochemically. Activity of ZOL on cholesterol biosynthesis was determined by measuring the incorporation of ^14^C mevalonate in cholesterol.

**Results:**

ZOL induced dose-dependent inhibition of proliferation of all the three cell lines although it appeared more efficient on the untransformed PNT1A. Whatever the cell line, 20 μM ZOL-induced inhibition was reversed by geranyl-geraniol (GGOH) but neither by farnesol nor mevalonate. After 48 hours treatment of cells with 20 μM ZOL, geranyl-geranylation of Rap1A was abolished whereas farnesylation of HDJ-2 was unaffected. Inhibition of Rap1A geranyl-geranylation by ZOL was rescued by GGOH and not by FOH. Indeed, as observed with treatment by a geranyl-geranyl transferase inhibitor, treatment of PNT1-A cells with 20 μM ZOL prevented the LPA-induced formation of stress fibers. We checked that in vitro ZOL did not inhibit geranyl-geranyl-transferase I. ZOL strongly inhibited cholesterol biosynthesis up to 24 hours but at 48 hours 90% of this biosynthesis was rescued.

**Conclusion:**

Although zoledronic acid is currently the most efficient bisphosphonate in metastatic prostate cancer management, its mechanism of action in prostatic cells remains unclear. We suggest in this work that although in first intention ZOL inhibits FPPsynthase its main biological actitivity is directed against protein Geranylgeranylation.

## Background

Bisphosphonates (BPs) have been used in oncological practice for many years to reduce skeletal complications and pain especially during myeloma and the metastatic phase of breast and prostate cancers. For many years their specific bone targeting suggested that the main mechanism of action of BPs was inhibition of bone resorption by direct effects on osteoclasts or other bone cells in the immediate microenvironment of osteoclasts[1]. The late-generation BPs have a nitrogen-containing aliphatic side chain (pamidronate, alendronate, ibandronate) or heterocyclic ring (zoledronate). Their anti-resorptive potency is up to 1000-fold greater than that of non-amino BPs and they exert their cellular effects by interference with the mevalonate (MVA) pathway [2]. Many studies have investigated the nature of the enzymatic targets of N-BPs within the MVA pathway. In vitro, squalene synthase activity was shown to be affected by some N-BPs but neither by pamidronate nor alendronate[3]. More recently, farnesyl-pyrophosphate (FPP) synthase was proposed as the main enzymatic target of alendronate, risedronate, ibandronate, and pamidronate in vitro [4-6]. Some authors suggested that isopentenyl (IPP) isomerase could also be inhibited by novel N-BPs [4,5]. In vivo, some studies confirmed the hypothesis of an inhibition of FPP synthase because apoptosis [7-9] and caspase activation [7] induced by several N-BPs were reversed by addition of FPP and GGPP or of cell-permeable analogs, farnesol (FOH) and geranyl-geraniol (GGOH). Conversely, recent in vivo studies suggested that the inhibition of the mevalonate pathway by N-BPs lies downstream the FPP synthesis step; indeed, several cellular effects of N-BPs such as inhibition of osteoclast formation[10], inhibition of ovarian cancer cell migration [11] or inhibition of breast cancer cell invasion [12] were reversed only by GGOH but not by FOH. Whatever the enzymatic target(s) of N-BPs most previous reports agree to suggest that the action of N-BPs results in the impairment of protein isoprenylation in osteoclasts or bone explants [4,5,8,10,13,14] as well as in tumoral cell lines [9,12,15-17]. It remains unclear whether protein farnesylation and geranyl-geranylation are equally affected by N-BPs. Some recent data suggest that geranyl-geranylation, especially of Rho GTPases, may be the main target of their anti-invasive effect [11,12] although their apoptotic effect may be related to the inhibition of Ras farnesylation [16,17].

In prostate tumoral cells, few studies have investigated the cellular or molecular effects of N-BPs, while zoledronate (ZOL) and pamidronate (PAM) are widely used to prevent skeletal complications of prostate cancer and of androgen deprivation therapy [18,19]. After the report of the inhibition of prostate carcinoma cell invasion by several N-BPs, including ZOL [20], PAM and ZOL were shown to inhibit growth in three widely used prostatic cell lines, PC-3, DU-145 and LNCaP [21]. The anti-tumor effect of alendronate, ZOL and PAM was correlated to their inhibition of the MWA pathway in prostate cells [15,17], favoring the hypothesis of an inhibition of FPP synthesis, protein geranyl-geranylation was not assessed although GGOH appeared more effective than FOH to rescue ZOL-induced apoptosis.

In the present work, we investigated i) the effect of ZOL and MVA-derived metabolites on tumoral and normal immortalized prostatic cell line proliferation; ii) the modification by ZOL of the prenylation status of farnesylated (HDJ-2) and geranyl-geranylated (Rap1A) proteins in prostatic cells; iii) the impact of ZOL on the biological activity of a geranyl-geranylated protein, RhoA; vi) the effect of ZOL on cholesterol biosynthesis.

## Methods

### Chemicals and materials

All material for cell culture was from Dutcher (Brumath, France). Growth media were purchased from Cambrex Bio Science (Verviers, Belgium) and fetal calf serum (FCS) from Invitrogen (Cergy-Pontoise, France). Zoledronic acid [hydrated disodium salt of 2-(imidazol-1-yl)-hydroxyl-ethylene-1,1-bisphosphonic acid)] was supplied by Novartis Pharma AG (Basel, Switzerland). Stock solutions (4 mM) were prepared in phosphate buffered saline (PBS) and aliquots were stored at -20°C to be diluted in culture medium prior to experiments. Peptidomimetic prenyl-transferase inhibitors FTI 277 and GGTI 298 [22,23] were a generous gift from Pr S. Sebti (Tampa, Florida). Stock solutions (100 mM) were prepared in DMSO-10 mM DTT and aliquots were stored at -20°C to be diluted in culture medium prior to experiments. Y27632 was purchased from Calbiochem (La Jolla, California). Mevalonate (stock = MVA 200 mM), trans-transfarnesol (FOH; stock solutions 100 mM in ethanol, stored at -20°C), geranyl-geraniol (GGOH; stock solutions 100 mM in ethanol stored at -20°C), squalene (stock solutions 100 mM in ethanol stored at -20°C FOH), Cholesterol, L-α- lysophosphatidic acid (LPA) and paraformaldehyde (PFA) were purchased from Sigma Aldrich (St Quentin Fallavier, France). [^14^C]-mevalolactone (56Ci/mmol. specific activity) was from Perkin-Elmer.

### Prostatic cell lines

LNCaP cells are prostatic tumoral human cells expressing an active but mutated (T877A) androgen receptor. Under androgenic stimulation, these cells produce prostate-specific antigen (PSA). PC-3 are prostatic tumoral human cells, which are non-responsive to androgenic stimulation. The PNT1-A cell line was developed by Cussenot et al. [24]. The cells are normal epithelial cells immortalized by SV-40. They express the wild-type androgen receptor but, under androgenic stimulation in vitro, they do not secrete PSA into the culture medium. The three cell lines were purchased from the European Collection of Cell Culture (PC-3: # 90112714; LNCaP clone FCG: # 89110211; PNT1A: #95012614).

Prostatic cell lines were routinely grown either in RPMI 7% FCS (PC-3 and PNT1-A) or in RPMI 10% FCS supplemented by 10 mM Hepes and 1 mM sodium pyruvate (LNCaP). All cells were incubated at 37°C in a humidified 5% CO_2 _incubator.

### Proliferation colorimetric assay

Proliferation was assessed by a colorimetric assay with sulforhodamine as described by Skehan [25]. Cells were seeded in 96-well microtiter plates to finally obtain 80% confluence in control wells. At the end of the treatment period, the cells were fixed with TCA (1 h, 4°C) and then stained with 0.4 % sulforhodamine. After four washes with 1% acetic acid to remove unbound dye, the plates were dried and protein-bound dye was extracted with Tris (10 mM, pH = 10.5). Coloration was quantified at 540 nm in a microtiter plate (Multiskan^® ^Multisoft, Labsystems)

### Prenylation status analysis

Cells were scraped off then lysed with 150 μL of lysis buffer [20 mM Tris-HCl, 100 mM NaCl, 1% Triton X-100, 10 mM MgCl_2_, 5 mM EDTA, 20 mM NaF, 10 mM PNPP, 2 mM Na orthovanadate, and a cocktail of protease inhibitors (Sigma, France, final dilution 1/100). Proteins were quantified in cellular extracts by a Bradford assay. Five to forty μg of cleared protein extracts were separated on a 12.5% SDS-polyacrylamide gel and then transferred to a Hybond-P polyvinyldifluoride membrane (Amersham Biosciences). After pre-incubation with TBST (25 mM Tris, 140 mM NaCl, 0.1% Tween 20, pH 8) supplemented with 5% fat-free milk, the membranes were incubated overnight at 4°C with the primary antibody diluted in TBST 1 % milk. After three washes with TBST, the membranes were incubated for 1 hour at room temperature with the secondary antibody labeled by peroxidase. The antibody was visualized using the ECL Plus system (Amersham Biosciences) and luminescence quantified with Phophorimager^® ^(Molecular Dynamics).

The following primary antibodies were used: anti-HDJ2 Ab-1 mouse monoclonal antibody (Interchim; clone KA2A5.6, diluted 1/10000), anti-prenylated Rap1A C17 goat polyclonal antibody (TEBU; ref SC-1482, diluted 1/2000), anti-total Rap1A-Krev1 rabbit polyclonal antibody (TEBU; ref SC-65, diluted1/1000). The following peroxidase-labeled secondary antibodies were used, all diluted 1/10000: anti-mouse (Biorad, ref 170–6516) anti-goat (Santa Cruz, ref sc-2033) or anti-rabbit (Biorad, ref 170–6515).

### Visualization of actin cytoskeleton by fluorescence microscopy

At day 0 (D0), PNT1-A cells were seeded onto glass coverslips in 6-well plates to obtain 60% confluence on day 3 (D3). On day 1 (D1), the cells were serum-starved until treatment on D3. After exposure to different drugs and then after stimulation by LPA (20 μM, 5 hours on D3), the cells were fixed with 3% paraformaldehyde/PBS for 20 minutes and then permeabilized with 0.1% Triton- X-100/PBS for 5 minutes. To visualize the actin fibers, the coverslips were incubated with tetramethylrhodamine isothiocyanate-labeled phalloidin (Molecular Probes) for 30 minutes at room temperature. The cells were viewed on a Zeiss Axiophot microscope and pictures taken with a Princeton camera.

### In vitro farnesyl-transferase (FTase) and geranyl-geranyl-transferase I (GGTase I) activity measurement

To investigate the putative inhibitory effect of ZOL on prenyl-transferase activity, in vitro prenylation assays were performed. As substrate proteins, we used bacterially expressed wild-type H-Ras protein for FTase assay and CVLL-H-Ras mutant protein (geranyl-geranylated form) for GGTase I assay. 1 μM substrate proteins were incubated with [^3^H]-prenyl pyrophosphate (0.25 μM final concentration, specific activity 8–10 Ci/mmol, American Radiolabeled Chemicals) and either recombinant FTase or recombinant GGTase I (50 ng) in a reaction medium containing 50 mM Tris, pH 7.7, 20 mM KCl, 10 mM MgCl_2_, 2 mM DTT, and 5 μM ZnCl_2_. The reaction volume was 50 μl. Reactions proceeded for 10 min at 30°C and were stopped by the addition of 0.5 ml of 4% SDS. Total protein was precipitated by the addition of 0.5 mL of 30% trichloroacetic acid. After 20 min, samples were filtered through 25-mm glass fiber filters (Schleicher & Schuell), which bound the prenylated protein leaving unprenylated protein and excess prenyl groups to go through the filter. Reaction tubes were washed with 2 × 2 mL of 4% SDS plus 6% trichloroacetic acid, and the washes were added to the filters. Bound protein was washed with a further 4 mL of 4% SDS/6% trichloroacetic acid and then 3 × 2 mL 6% trichloroacetic acid. After drying, radioactivity bound to the filters was counted in a scintillation counter. Negative controls were also performed without protein substrate or using enzymes that were previously inactivated by incubation for 5 min at 90°C. Each substrate protein was reacted under standard conditions with vehicle, specific transferase inhibitor or ZOL. The level of prenylation was expressed as a percentage of maximum incorporation of [^3^H]-prenyl for each substrate, as determined by allowing the uninhibited reaction to go to completion.

### [^14^C]-Cholesterol biosynthesis in PC3 cells

The experiment was conducted as described by Awad [26]. Briefly, PC3 cells were seeded in 6-well plates and treated with ZOL for 2, 22, 46 hours and [^14^C]-mevalolactone (2.25 μCi/ml) was added to the medium for 2 hours more, then the medium was removed, the cells washed twice with PBS, and treated by 1 ml of NaOH (2 M). The mixture was incubated at 37°C for 30 min. and then 0.5 ml of methanol containing 100 μg of non-radioactive cholesterol were added. Saponification took place at 70°C for 1 hour and unsaponified lipids were hexane extracted three times. After drying (under argon) the pellets were dissolved in chloroform and separated by thin layer chromatography (silica gel F/ethyl acetate). [^14^C]-Cholesterol was revealed and quantified by autoradiography with Phophorimager^® ^(Molecular Dynamics).

## Results

### Zoledronic acid inhibits proliferation of LNCap, PC-3 and PNT1-A cells

**Figure 1:**
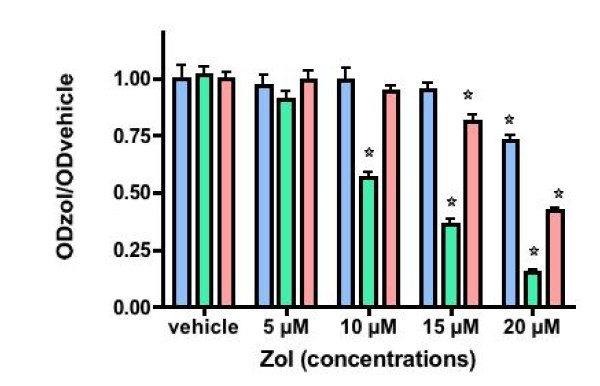
**Effect of zoledronate on proliferation of PC-3, PNT1-A and LNCaP**. At D0, the cells were seeded into 96-well plates to obtain 80% confluence at D5 in control wells. At D1 and D3, cells were treated by vehicle or increasing doses of zoledronate (ZOL: 5, 10, 15, 20 μM). At D5, the cells were fixed with TCA and stained with 0.4% sulforhodamine. Staining intensity was quantified at 540 nm. Results are expressed as the ratio OD_treatedcells_/OD_untreated _of three independent assays each performed six times. Error bars indicate inter-assay mean ± 1 SD. * indicates a significant difference versus non-treated cells (p < 0.01).  PC3  PNT1A  LNCaP

The effect of ZOL on cell growth was assessed in the three prostatic cell lines: the cells were treated from D1 to D5 with ZOL from 5 to 20 μM. The ratio of proliferation versus control wells was assessed by a colorimetric sulforhodamine assay. The results are shown in figure 1. ZOL inhibited the proliferation of the three cell lines dose-dependently. The non-tumoral cell line PNT1-A was affected most (IC_50 _= 11 μM). PC-3 cells displayed an intermediate sensitivity (IC_50 _= 18 μM) and the LNCaP cells appeared to be the most resistant (IC_50_>20 μM). These results agree with those previously obtained by others for growth [21,27] or viability [15] of DU145, PC-3 and LNCaP in the presence of ZOL. Moreover the present work shows that ZOL is more effective at inhibiting the cell growth of the untransformed cell line PNT1-A.

### Inhibition of proliferation by ZOL is reversed by geranyl-geraniol

**Figure 2:**
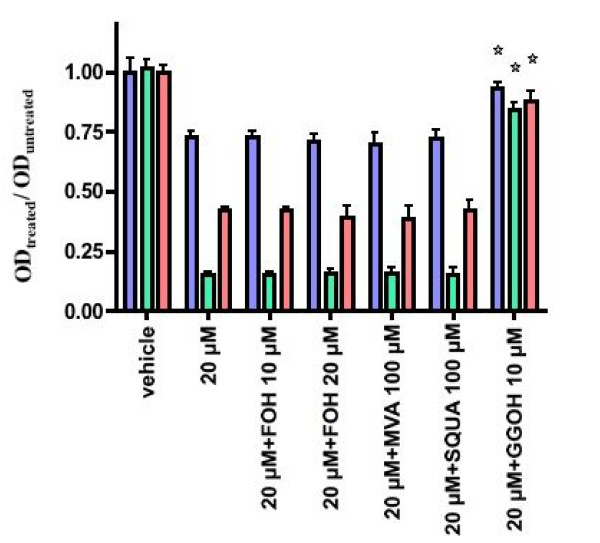
**Effect of mevalonate-derived products on 20 μM zoledronate-induced inhibition of prostatic cell proliferation**. At D0, cells were seeded into 96-well plates to obtain 80% confluence at D5 in control wells. At D1, and D3, cells were treated by vehicle or 20 μM zoledronate (ZOL) in the presence or absence of intermediates of the mevalonate pathway (GGOH = geranyl-geraniol; MVA = mevalonate; FOH = farnesol; SQUA = squalene) at the indicated doses. Results are are expressed as the ratio OD_treated cells_/OD_untreated _of three independent assays each performed six times. Error bars indicate inter-assay mean ± 1 SD. * indicates a significant difference versus ZOL-treated cells (p < 0.01).  PC-3  PNT1A  LNCaP

N-BPs have been described to interfere with the mevalonate pathway. We therefore investigated if the inhibition of proliferation observed with 20 μM ZOL could be reversed by metabolites of the MVA pathway: MVA (100 μM), the precursor of all sterol and non-sterol isoprenoids, squalene (SQUA, 100 μM), the precursor of cyclic isoprenoids i.e. sterols, farnesol (FOH, 10 and 20 μM) and geranylgeraniol (GGOH, 10 μM). Unlike FPP and GGPP, free isoprenols FOH and GGOH are able to enter the cells easily [28], thus, they can be used for prenylation via a salvage pathway. Cells were seeded as previously described and treated every 48 hours with 20 μM ZOL in the presence or absence of the different metabolites from D1 to D5. The results are shown in figure 2. Concerning the differential sensitivity of the three cell lines to ZOL, the results were similar to those previously obtained although a two-day longer exposure resulted in a greater cell growth inhibition with 20 μM ZOL. At the concentrations used, none of the MVA-derived products altered the proliferation (data not shown). Moreover, neither MVA, SQUA, or FOH could reverse the inhibition of proliferation induced by 20 μM ZOL. In contrast, GGOH completely rescued the proliferation of the three prostatic cell lines when added to the culture medium simultaneously with 20 μM ZOL. However, the reversion of ZOL-induced inhibition of proliferation by GGOH but not by FOH does not agree with the biochemical target of ZOL, FPP synthase.

### ZOL impairs in vivo geranyl-geranylation of Rap1A without impairing farnesylation of HDJ-2

**Figure 3:**
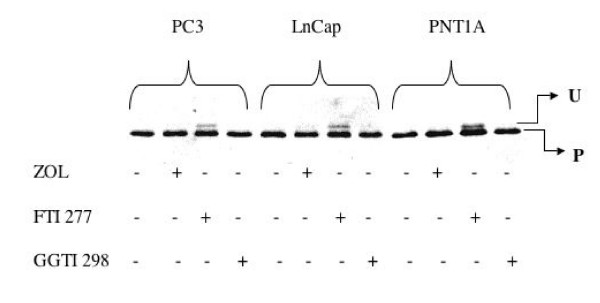
**Effect of zoledronate on farnesylation status of HDJ2**. Prostatic cells where treated for 48 h with ZOL (20 μM), lysed in hot (90°C) SDS (1%) and analyzed by Western blot.

To investigate the effects of ZOL on farnesylation and geranyl-geranylation, we analysed i) Rap-1A, an exclusively geranyl-geranylated small GTPase involved in the regulation of signal transmission from Ras, ii) HDJ-2, an exclusively farnesylated co-chaperone protein classically used as a marker of FTase inhibition [29]. Western-blot analysis of these proteins was performed after treatment by 20 μM ZOL, or by specific peptidomimetic inhibitors of farnesylation (FTI-277) or geranyl-geranylation (GGTI-298). The results are shown in figure 3. Treatment by FTI 277 (10 μM, 48 hours) resulted, as expected, in the accumulation of the unfarnesylated HDJ-2 which migrates slower than the farnesylated forms. By contrast, treatments with GGTI-298 or ZOL (20 μM, 48 hours) did not modify HDJ-2 farnesylation. At this point we questioned the in vivo efficacy of ZOL on the mevalonate pathway and decided to look for its effects on protein geranyl-geranylation.

### ZOL impairs Rap1A geranyl-geranylation by preventing GGPP production and not GGPP incorporation into the protein

**Figure 4:**
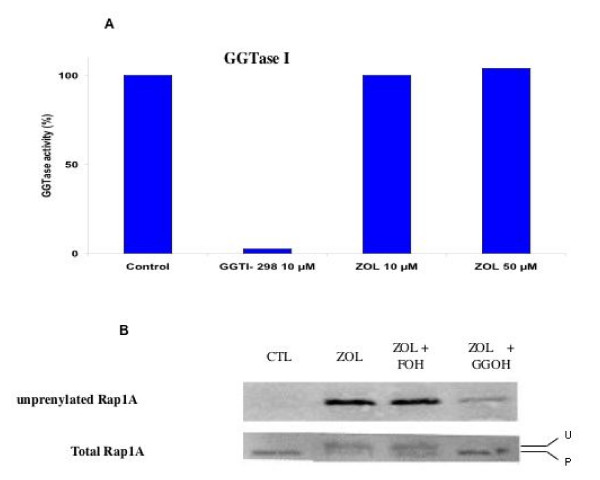
**Effect of zoledronate on GGPP/GGOH incorporation into proteins in vitro (A) and in vivo (B)**. **A**: Geranyl-geranyl transferase I (GGTase I) was quantified using an *in vitro *test based on [^3^H]-prenyl pyrophosphate (0.5 μM, 8–10 Ci/mmol) incorporation into a mutant form of H-ras with a geranyl-geranylation CAXX box. The level of prenylation is expressed as a percentage of maximum incorporation of [^3^H]-prenyl, as determined by allowing the uninhibited reaction to go to completion. **B**: Western-blot analysis; PC-3 cells are treated by vehicle, ZOL 20 μM; ZOL 20 μM + FOH 10 μM; ZOL 20 μM + GGOH 10 μM.

Indeed, there is a structural analogy between the bisphosphonate radical of ZOL and the pyrophosphate group of cell substrates of FTase and GGTases, respectively FPP and GGPP, and some bisphosphonate analogues of FPP were shown to inhibit FTase [30]. To challenge these two hypotheses, we tested the ability of ZOL to prevent GGPP incorporation into substrate proteins in vitro and in vivo. The results are shown in figure 4. In vitro, we measured GGTase I activity, in the absence or presence of 10 and 50 μM ZOL. Unlike GGTI-298, which totally abolishes GGTase I activity, ZOL did not impair the enzymatic activity of GGTase I (figure 4A). We also checked that ZOL did not impair the enzymatic activity of FTase (data not shown). In vivo, we used GGOH as a surrogate for GGPP because it has been shown that GGOH can rescue Rap1A geranyl-geranylation in lovastatin-treated cells, [31]. In prostatic cells, rescue of Rap1-A geranyl-geranylation was obtained when 10 μM GGOH was added simultaneously to 20 μM ZOL. This recovery was observed neither after ZOL/FOH incubation, nor after GGTI/GGOH incubation (figure 4B).

So, inhibition of geranyl-geranylation by ZOL appears to result from a blockade in GGPP synthesis from FPP rather than from the inhibition of the enzymatic transfer of GGPP onto its protein substrate.

### ZOL impairs LPA-induced formation of stress fibers, a cellular effect dependent on RhoA geranyl-geranylation

**Figure 5:**
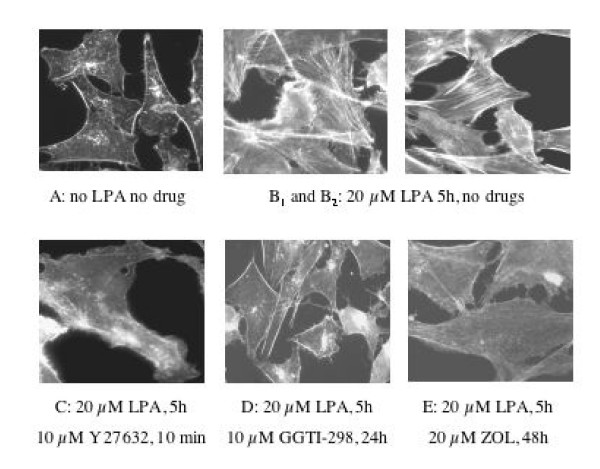
**Effect of zoledronate on LPA-induced stress fiber induction, a cellular effect dependent on RhoA geranylgeranylation**. On D_0_, PNT1-A cells were seeded onto glass coverslips in 6-well plates to obtain 60% confluence on D_3_. On D_1_, the cells were serum-starved till fixation on D_3_. After treatment by the indicated drugs then stimulation by LPA (20 μM, 5 hours on D_3_), actin fibers were visualized by tetramethylrhodamine isothiocyanate-labeled phalloidin. Cells are viewed on a Zeiss Axiophot microscope (X 630), and pictures are taken with a Princeton camera.

RhoA is a small GTPase, which is exclusively geranyl-geranylated. RhoA geranyl-geranylation is crucial for its biological activity, especially for its specific activity in the formation of stress fibers under LPA stimulation [32]. For this reason, we investigated the effect of ZOL on the formation of stress fibers induced by LPA stimulation. Among the three prostatic cell lines, only PNT1-A cells form an easily detectable stress fiber network in response to LPA. So, we chose this cell line for the experiment. PNT1-A cells were cultured as described above and stimulated by 25 μM LPA for 5 hours. Under these conditions, numerous stress fibers appeared (fig 5B1 and 5B2). With pre-treatment by Y27832 (10 μM, 10 min), a specific Rho kinase inhibitor, stress fiber induction was totally abolished (figure 5C). Similarly, when PNTI-A cells were treated with GGTI-298 (10 μM, 24 hours) prior to LPA stimulation, no stress fibers appeared (figure 5D). When PNT1-A cells were treated by ZOL (20 μM, 48 hours) prior to LPA stimulation, stress fiber formation was also totally abolished (fig 5E). So, we suggest that inhibition of geranyl-geranylation by ZOL affects the biological activity of geranyl-geranylated proteins, as shown herein for the small GTPase RhoA.

### ZOL Inhibits cholesterol biosynthesis up to 24 hours

**Figure 6:**
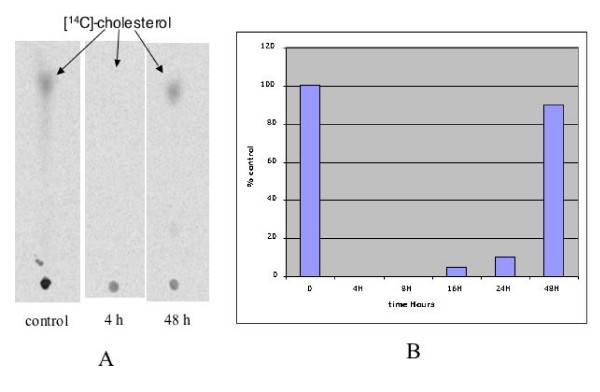
**Biosynthesis of [^14^C]-cholesterol in PC3 cells under ZOL treatments**. Cells were treated with ZOL (20 μM) for 2, 22 or 46 hours and [^14^C]-mevalolactone (2.25 μCi/ml) was added to the medium for 2 hours more. Sterols were separated by thin layer chromatography (silica gel F/ethyl acetate). [^14^C]-Cholesterol was revealed and quantified by autoradiography with Phophorimager^® ^(Molecular Dynamics).

Four hours treatment of PC3 cells with ZOL 20 μM completely inhibited cholesterol biosynthesis (Fig 6A) suggesting that ZOL, as previously reported, may inhibit farnesyl pyrophosphate synthase at the crossroads of the mevalonate pathway to cholesterol biosynthesis or protein isoprenylation. However, as early as 24 hours later, about 10% of the biosynthesis was rescued, reaching 90% after 48 hours (Fig 6). The same results were obtained with LnCAP and PNT-1A (data not shown).

## Discussion

Although zoledronic acid is widely used in metastatic prostate cancer management, few data are available about its molecular effects in prostatic cells [15,20,21]. Our work shows that, in prostatic cells, ZOL impairs protein geranyl-geranylation but not farnesylation. This result contrasts with the well-documented in vitro inhibition of FPP synthase displayed by many N-BPs [4-6,33] including ZOL [33]. However, conflicting results about the FPP synthase inhibition by N-BPs have been obtained in vivo. Indeed, some studies support the inhibition of FPP synthase, impairing FPP, and consequently GGPP, production [7-9,15,17], whereas other teams suggest the existence of an additional enzymatic target of N-BPs, downstream of FPP synthase [11,12,34]. Our results with ZOL lend further support to this latter hypothesis and corroborate two other works performed in mammary MDA-MB-231 [12] and ovarian Caov-3 [16] tumoral cell lines. In the first report [12], it was shown that the anti-invasive effect of ZOL resulted from an inhibition of RhoA geranyl-geranylation. In alendronate-treated Caov-3 cells, Sawada et al. showed inhibition of Rho activation through GGPP depletion and suggested that this inhibition accounted for the inhibition of LPA-induced stress fiber formation they observed. This biological effect is typically related to RhoA activity.

## Conclusion

In the present work it is shown that the main biological activities of ZOL are sustained by its inhibitory effect of protein geranyl-geranylation. We show by study of cholesterol biosynthesis that this impairment of geranyl-geranylation is time-dependently related to an inhibition of FPPsynthase followed by, or concomitant with, inhibition of GGPPsynthase. In vitro studies show that this effect is not related to inhibition of GGTase I

## Competing interests

The author(s) declare that they have no competing interests.

## Authors' contributions

The authors' contibutions to this research are reflected in the order shown, with the exception of SSV who supervised the research and the preparation of the manuscript. MG and MT carried out cellular biology and CW enzymatic studies. AP, ILM provided helpful advices in immuno-studies, JCF and SSV conceived of the study and participated in its design and coordination. All the authors read and approved the final manuscript

## Pre-publication history

The pre-publication history for this paper can be accessed here:
